# Selection, optimization and compensation strategies and their relationship with well-being and impulsivity in early, middle and late adulthood in a Polish sample

**DOI:** 10.1186/s40359-021-00650-2

**Published:** 2021-09-16

**Authors:** Ludmiła Zając-Lamparska

**Affiliations:** Faculty of Psychology, Kazimierz Wielki University, ul. Staffa 1, 85-867 Bydgoszcz, Poland

**Keywords:** Selection, Optimization and compensation model, Successful development, Well-being, Impulsivity, Adulthood

## Abstract

**Background:**

The model of selection, optimization, and compensation (SOC model) characterises the life management strategies that contribute to successful development. Although the SOC model is an important developmental theory, until now there has been no Polish version of a measurement tool for SOC strategies. The studies conducted so far have consistently indicated a relationship between the use of these strategies and well-being. In contrast, the relationship between SOC strategies and impulsivity has not yet been empirically examined, although there are theoretical premises to assume its existence. The aim of the study was to compare the use of SOC strategies in early, middle and late adulthood, and to investigate the relationship between the use of SOC strategies and well-being and impulsivity, using a newly developed Polish version of the SOC Questionnaire (SOC48-PL).

**Methods:**

The study applied a quantitative approach. The sample comprised 589 Poles from three age groups: early adulthood (n = 186, 20–35 years), middle adulthood (n = 165, 40–55 years) and late adulthood (n = 238, 60–85 years). In the study, in addition to the SOC48-PL questionnaire, the Short Depression-Happiness Scale (SDHS) and the Short UPPS-P Impulsive Behavior Scale (S UPPS-P) were used.

**Results:**

Developmental differences in the use of SOC strategies are rather slight, with considerable individual differences within age groups. The study revealed some indications of a ‘developmental peak’ of SOC strategies in middle adulthood. The use SOC strategies is positively related to well-being. In turn, the relationship between the use of SOC strategies and impulsivity is negative, especially for two dimensions of impulsivity: lack of premeditation and lack of perseverance.

**Conclusions:**

The results only slightly confirm the assumed age-related differences in the use of SOC strategies and point to a large role of individual differences. The revealed positive relationships of SOC strategy use with well-being and negative with impulsivity are consistent with expectations. With the present study, the SOC48-PL questionnaire may find application in further studies on the SOC model involving the Polish population, as well as in the measurement of SOC strategies in psychological practice. Furthermore, the identified associations of SOC strategies with well-being and impulsivity can be taken as initial indications for the development of interventions based on teaching the use of SOC strategies. Cross-cultural comparisons, long-term longitudinal studies on changes in the use of SOC strategies over the life course, and combining quantitative and qualitative approaches in the analysis of the use of SOC strategies in practice are worth mentioning as directions for further research.

**Supplementary Information:**

The online version contains supplementary material available at 10.1186/s40359-021-00650-2.

## Introduction

Contemporary reality offers a wide range of developmental directions and trajectories, therefore the opportunities for managing one’s own development have to be skilfully used. Considering this phenomenon, understanding the psychological mechanisms of successful life-management and the possibility of measuring them gains special significance. A psychological model describing the strategies that are used for life-management during life span is the model of selection, optimization, and compensation (SOC model) by Baltes and Baltes [1]. The SOC model is a metatheory referring to successful development. The SOC strategies serve to adjusting the course of development by initiating, maintaining and terminating actions related to the achievement of goals [1–3].

An instrument measuring strategies described by this model is the SOC-Questionnaire (Selection, Optimization, and Compensation Questionnaire), which was originally created in the German language, and translated into English by its authors [4]. Besides these two versions, four further language versions have been developed to date [5–8]. So far, however, no Polish version has been developed that would allow study of SOC strategies in the Polish population.

The SOC model was created as a conceptualisation of successful aging, however, practically at once it was recognised that the model is relevant to all stages of development [1, 2, 9, 10]. In light of the SOC model, developmental changes over the life span in terms of the use of SOC strategies are postulated. At the same time, there are two different hypotheses as to the shape of the trajectory of these changes—either an increasing trend is assumed, or first an increasing and then a decreasing trend [1, 3]. Moreover, somewhat surprisingly, not many studies have examined age differences in the use of SOC strategies, which makes studies on this issue worthwhile.

The authors of the SOC model assume that the strategies described in it are valid predictors of well-being, as a subjective indicator of life-management and successful development [3, 11]. Although the SOC model was developed quite a long time ago [1, 2], the association between SOC strategies and well-being has been repeatedly confirmed by research over the years [e.g., 11–15]. Faced with the constantly changing contexts of human existence, it is worth to demonstrate the continuously positive role of SOC strategies in various groups of persons.

The importance of SOC strategies is also reflected in their associations with other psychological variables, such as personality and emotional and mental characteristics [3, 16–18]. However, so far, there has been no study on the association of the SOC strategy use with impulsivity, although the SOC model provides rationales for their negative relationship [1, 2]. Moreover, given that the use of SOC strategies can be effectively trained [19, 20], the evidence of a link between impulsivity and SOC strategies may, in the future, result in research into the potential for coping with the negative consequences of impulsivity using SOC strategies.

The objectives of the present study are defined in the context of the above brief discussion. The study was aimed to assess the SOC strategies in Polish adults, using a newly developed Polish version of the SOC Questionnaire—SOC48-PL, to compare the use of SOC strategies in three age groups: early, middle and late adulthood, and to evaluate the relationship between the use of SOC strategies and well-being and impulsivity, also in these age groups.

The present study posed the following research questions:

1. Is the Polish version of the SOC Questionnaire (SOC48-PL) reliable?
2. Are there differences between individuals in early, middle and late adulthood in terms of the use of SOC strategies?
3. Is there a relationship between the use of SOC strategies and well-being in individuals in early, middle and late adulthood?
4. Is there a relationship between the use of SOC strategies and impulsivity in individuals in early, middle and late adulthood?

The study applied a quantitative, correlational approach. A cross-sectional model was used to evaluate age-related differences.

## Literature review

### The SOC model and life-management strategies

The model of selection, optimization, and compensation (SOC) relies on the action theoretical approach [1]. According to it, the use of life-management strategies enables individuals to function effectively in changing circumstances, through the optimised use of limited resources, consequently leading to successful development, and ensuring subjective well-being [1–3, 21]. The SOC model describes three strategies of life-management: selection (S), optimization (O), and compensation (C), and two categories have been distinguished for selection: elective selection (ES) and loss-based selection (LS) [3]. All these strategies are used for life-management and adjusting the course of development by initiating, maintaining and terminating actions related to the achievement of goals [1, 3]. Both aspects of selection relate to goal setting. Elective selection aims at achieving desired states in specific areas of functioning. Thus, this strategy sets the direction for development. Loss-based selection occurs in a situation of real or expected loss (or reduction) of resources which requires the adjustment of the posed goals. Optimization and compensation strategies relate to achieving goals. The use of one of these two strategies depends on the availability of resources. Optimization refers to the acquisition and investment of internal and external resources that are essential for the achievement of selected goals. Finally, compensation is a strategy involving the use of alternative resources and the choice of alternative methods to achieve a given goal, if the resources and methods used thus far are temporarily or permanently unavailable [3]. Individual strategies of the SOC model form an interactive and cooperative system, to which the authors refer by using the term ‘orchestration’ [2, 22, 23].

### Measurement of the SOC strategies

An instrument measuring strategies described by the SOC model is the SOC-Questionnaire (Selection, Optimization, and Compensation Questionnaire), which was originally created in the German language, and translated into English by its authors [4]. The questionnaire includes 4 scales, measuring the tendency to use 4 strategies: Elective selection (ES), Loss-based selection (LS), Optimization (O) and Compensation (C). There are two versions of this instrument: long (48 items, 12 items per scale) and short (12 items, 3 per scale) [3, 4]. So far, in addition to the original versions, four versions of the SOC-Questionnaire have been created. In chronological order these are: Chinese [5], Swedish [6], Japanese [7] and Spanish [8] versions. New versions of the SOC-Questionnaire were found to have satisfactory psychometric parameters and proved to be adequate for measuring SOC strategies among respondents using the relevant languages. Because of these promising findings and the usefulness of the SOC-Questionnaire in research on successful development, it seemed valuable to develop a Polish version of this instrument to provide an entry point for research on the use of SOC strategies in the Polish population.

### Developmental changes in the use of SOC strategies over the life span

According to the SOC model, the use of SOC strategies shows developmental dynamics, which is associated with the changing context of the individual's life [3, 16, 24, 25].

For adulthood, there are two different theoretically justifiable hypotheses regarding the developmental trajectory of SOC [3]. According to the first of these, as one grows older, one becomes better at using SOC strategies thanks to the accumulation of life experiences [1]. In turn, according to the second one, the biological and physical constraints associated with aging imply a losses of resources, which may limit the use of SOC strategies. As a consequence, in old age the incidence of SOC-related behaviours might decline [3]. This suggests a developmental trajectory in which the use of SOC strategies first increases with age and then decreases.

Empirical data on the developmental dynamics of the SOC strategies come from cross-sectional studies, which actually are not very numerous. According to these data, the developmental peak of SOC occurs in middle adulthood, when the tendency to use most of the SOC strategies is highest, compared to early and late adulthood. Only the use of elective selection increases steadily with age, from early to late adulthood. In addition, persons in middle adulthood show the highest consistency for SOC components [3]. The decline in the use three of the SOC strategies between middle and late adulthood is associated with the depletion of resources, as assumed by the second hypothesis mentioned above. However, older adults can compensate for this by more efficient use of these strategies [26, 27]. Ageing is also associated with the shift of motivation, from striving for gains to maintenance and prevention of losses [21, 28]. For this reason, loss-related strategies, such as compensation, can be more often used by older persons than younger ones [26].

There is a lack of longitudinal research that examines developmental changes in the usage of SOC strategies over the life span. If longitudinal studies on SOC strategies are conducted, they cover at most several months or several years of time. Moreover, these studies usually do not focus on developmental changes in SOC strategies themselves, but on the association of SOC strategies use with other variables, whose changes are examined [e.g., 12, 14, 25, 29, 30].

### The relationship between SOC strategies use and well-being

SOC strategies are treated as adaptive resources enabling effective life management and therefore the use of these strategies is related to successful development in many areas and in various age groups [e.g., 21, 29, 31–34].

In particular, a positive link between the use of SOC strategies and well-being, life satisfaction and positive affectivity, and a negative link with depressive tendencies, have been empirically demonstrated in many contexts, including: successful ageing and adaptation to age-associated losses and health problems [e.g., 11, 13, 19, 35–38], health behaviours, coping with stress and illness [e.g., 15, 29, 33, 34], occupational well-being and work-life balance [e.g., 16, 17, 20, 39, 40], and positive youth development—PYD [12, 41–43]. Constructs such as well-being, life satisfaction, positive affectivity, and depression in research on SOC strategies were measured in a variety of ways. However, the instruments applied most often measured either the positive (e.g., positive affectivity, life satisfaction) or the negative (e.g., depression) aspect of well-being. Another approach is to measure the happiness—depression continuum. Such a measurement is provided by the Depression-Happiness Scale (DHS) and its short version, the Short Depression-Happiness Scale (SDHS) [44]. However, to date these scales have not been used in research on SOC strategies.

### The relationship between SOC strategies use and impulsivity

So far, the relationship between the use of SOC strategies and impulsivity has not been investigated. However, on the theoretical grounds of the SOC model, its occurrence can be assumed [1, 2]. Considering the fact that the SOC model is treated as the conceptualisation of intentional self-regulation [45, 46] and as the life-span control model [47], it can be presumed that target-oriented SOC strategies are negatively associated with impulsivity, or at least some aspects of it. Currently, impulsivity is understood as a multi-faceted construct [48]. While some aspects of impulsivity are related to ongoing behaviour (motor/behavioural impulsivity/disinhibition/premature responding), others address self-control in a more global sense (cognitive impulsivity), including planning deficits (non-planning, decrease in orientation toward the future) and lack of persistence (problems with delay of gratification, termination of action before the goal is reached) [49–51]. And this second group of impulsivity aspects seems to be straightforwardly the opposite to the mechanisms underlying the SOC strategies. According to model of impulsivity based on factor analysis, impulsivity encompasses five dimensions: lack of premeditation (LoPrem), lack of perseverance (LoPers), negative urgency (NU) and positive urgency (PU), and sensation seeking (SS) [52]. To measure these dimensions the Urgency-Premeditation-Perseverance-Sensation Seeking-Positive Urgency (UPPS-P) Impulsive Behavior Scale and the short version of it (S UPPS-P) are used [53–55]. Lack of premeditation denotes the tendency to not reflect on the consequences of actions. Lack of perseverance is associated with self-discipline and refers to an individual's poor ability to remain focussed on a task that may be long, boring or difficult. Urgency indicates the tendency to experience strong impulses and act rashly under conditions of intense mood: negative (negative urgency) or positive (positive urgency). Sensation seeking refers to the tendency to seek out novel and thrilling experiences, including openness to taking risky activities [52–55]. Among these five dimensions of impulsivity, the hypothetical relationship with the SOC strategies seems to apply particularly to lack of planning for action (lack of premeditation—LoPrem) and lack of persistence in goals pursuit (lack of perseverance—LoPers). Planning is essential to basically all SOC strategies, both the selection of goals and their achievement. Elective selection (ES) requires goal setting and creating the system of goals and establishing their hierarchy. Loss-based selection (LS) is based on restructuring one’s hierarchy of goals and the search for new goals. Optimization (O) includes, among others, the ability to appropriate time allocation and resources, choosing the right moment of action and the use of modelling successful others. Finally, compensation (C) involves adjustments in the allocation of time and effort proportional to changes in resources [2, 3]. Lack of premeditation is contrary to these planning activities. In turn, perseverance is particularly necessary for the achievement of goals and therefore for optimization (O) and compensation strategies (C). Both of them require attentional focus, practice of skills, commitment and persistent effort [2, 3], which is in opposition to the lack of perseverance (LoPers). Moreover, impulsivity is known to be one of the most important predictors for risky behaviour and depression [48, 56–58], both of which are negatively related to SOC. A negative link of the use of SOC strategies with risky and problematic behaviour has been repeatedly reported, although studies on this topic were conducted largely among adolescents and young adults [12, 43, 59]. Although most studies on impulsivity are focussed on adolescence and early adulthood [e.g., 57, 58, 60, 61], impulsivity has also been shown to be important for various aspects of individuals' functioning in later developmental stages, including late adulthood. At the same time, the role of impulsivity may change during a life course [e.g., 62–66]. Therefore, the relationship between impulsivity and the use of SOC strategies may also change with age.

## Methods

### Participants

The sample size was predetermined basing on the a priori power analyses for comparison of the three age groups and correlation analyses. For all the analyses, the necessary sample size was computed as a function of the required power level (1 − β), the pre-specified significance level α, and the population effect size to be detected with probability of 1 − β [67]. For each analysis, a significance level of 0.05 was assumed, which is the typically adopted criterion, and a power level of 0.95, which exceeds the accepted minimum level of power of 0.80 [67, 68]. With regard to the comparison of age groups, on the basis of the results of previous studies, population medium effect size has been assumed [3, 26]. Effect sizes for the association between SOC and well-being strategies varied across studies [e.g., 3, 17, 20, 29, 38], therefore the medium population effect size has been assumed. For the association of SOC strategies with impulsivity, there is no previous research, so it was decided to adopt the same (medium) population effect size as for the other analyses. Following the criteria mentioned above, the required sample sizes have been calculated for (a) analyses aiming to compare the age groups, and (b) correlational analyses. The sample sizes required for each analysis were calculated according to the formulas implemented in G*Power [69].

According to the obtained results, for the three groups’ comparison in terms of five response variables (i.e., the largest number of response variables considered in this study), the total sample size should be a minimum of 201 participants for global effect of MANOVA and 252 participants for univariate ANOVAs.

As for correlation analyses, the calculated minimum sample size was 138 individuals. Given the division of the sample into age groups and conducting separate correlation analyses within them, each age group should include a minimum of 138 persons, resulting the required sample size of 414 participants.

The sample comprised 589 participants, including individuals in early adulthood (aged 20–35), middle adulthood (aged 40–55), and late adulthood (aged: 60–85). All participants were Polish citizens and used Polish as their native language. They were volunteers recruited in the snowball sampling procedure. Sample characteristics are provided in Table 1.

**Table 1:** Characteristics of the study participants

Age group	*N*	Age		Gender (%) women/men	Years of education	
		*M*	*SD*		*M*	*SD*
Early adulthood	186	25.06	3.97	58/42	15.42	1.82
Middle adulthood	165	47.62	4.33	64/36	14.36	2.89
Late adulthood	238	67.42	6.43	58/42	12.35	4.04

### Instruments

*SOC48-PL* is the Polish version of the SOC-Questionnaire (Additional file 1: SOC48-PL). It was created based on two original-language versions: German and English. The procedure of the SOC48-PL development is depicted in the Additional file 2: Fig. S1. The questionnaire consists of 48 test items, 12 items per scale: Elective selection (ES), Loss-based selection (LS), Optimization (O), and Compensation (C). Participants completing the questionnaire provide their answers in two steps. The first step is the same as for the original version of the SOC-Questionnaire, i.e., the participant is expected to indicate one out of two statements describing the behaviour of hypothetical individuals A and B that better represents him/her. In the second step, the participant is expected to clarify his/her answer using a 6-point graphical estimate scale and to specify how close his/her behaviour matches the behaviour of the previously indicated individual (A or B, respectively), given a choice of 3 degrees of similarity for each of the two behaviours. The answer given on the graphical scale is converted into a score in such a way that the more the respondent considers his/her own behaviour to be similar to the SOC strategy, the higher the score obtained. The reliability of the SOC48-PL was tested in the present study and is given in Table 2, and, in more detail, in Additional file 3: Table S1.

**Table 2:** Descriptive statistics of the measured variables in the three age groups and Cronbach's alpha coefficients of the measurement scales used in the study

Scale	Cronbach’s α	Early adulthood *M* (*SD*)	Middle adulthood *M* (*SD*)	Late adulthood *M* (*SD*)
SOC48-PL	0.93	148.54 (28.54)	151.46 (34.23)	145.58 (34.88)
ES	0.82	33.73 (9.70)	33.99 (10.92)	35.11 (10.93)
LS	0.81	36.09 (8.31)	38.10 (9.81)	35.85 (10.32)
O	0.86	40.76 (9.13)	41.21 (10.16)	38.51 (11.64)
C	0.83	37.95 (8.94)	38.16 (10.40)	36.11 (10.86)
SDHS	0.78	12.94 (3.35)	12.97 (3.07)	12.48 (3.14)
S UPPS-P	0.85	43.40 (7.75)	42.32 (8.02)	40.35 (8.39)
NU	0.78	9.13 (2.52)	9.55 (2.45)	9.62 (3.02)
PU	0.65	10.30 (2.30)	10.28 (2.27)	9.59 (2.51)
LoPrem	0.70	7.53 (2.07)	7.29 (2.15)	6.91 (1.95)
LoPers	0.82	6.80 (2.10)	6.85 (2.14)	6.49 (2.33)
SS	0.80	9.65 (2.65)	8.36 (2.74)	7.75 (2.75)

*SOC48-PL* SOC48-PL questionnaire, *ES* Elective selection, *LS* Loss-based selection, *O* Optimization, *C* Compensation, *SDHS* The Short Depression-Happiness Scale, *S UPPS-P* Short UPPS-P Impulsive Behavior Scale, *NU* Negative Urgency, *PU* Positive Urgency, *LoPrem* Lack of Premeditation, *LoPers* Lack of Perseverance, *SS* Sensation Seeking

*SDHS—The Short Depression-Happiness Scale* [44] was used to measure well-being defined as a continuum from happiness to depression. SDHS is a short 6-item instrument. The total score is in the range of 0–18. The higher the score obtained, the higher the level of well-being. Cronbach’s α coefficient for SDHS calculated in the analysed sample is provided in Table 2.

*S UPPS-P—The Short UPPS-P Impulsive Behavior Scale* [53] was used to measure 5 dimensions of impulsivity: negative urgency, positive urgency, lack of premeditation, lack of perseverance, and sensation seeking. Higher scores in the S UPPS-P indicate higher levels of impulsivity. Cronbach’s α coefficient for S UPPS-P calculated in the analysed sample is shown in Table 2.

### Data analyses

The reliability analysis for the SOC48-PL included (a) calculation of: Cronbach’s *α*, McDonald’s *ω* and Guttman’s *λ6* coefficients, correlations between individual items and the respective scale, as well as (b) the assessment of absolute stability in the test–retest procedure using Pearson's coefficient of correlation *r*. The use of SOC strategies, as well as impulsivity in the three age groups, was compared using one-way MANOVA test, supplemented with univariate ANOVAs and Bonferroni tests for multiple comparisons. For the well-being comparison, ANOVA was used. The relationship of the use of SOC strategies with well-being and impulsivity was assessed using the Pearson's coefficient of correlation *r*. All correlation analyses performed were supplemented with the False Discovery Rate (FDR) correction for multiple comparisons.

Statistical analyses were performed using Statistica 13 PL, JASP [70], and G*Power 3.1.9.4. [69].

## Results

### Reliability of the Polish version of the SOC-Questionnaire (SOC48-PL) in three age groups

The interpretations of the values of particular reliability coefficients adopted in the literature are various [71, 72], but the values obtained in these studies demonstrated that the SOC48-PL is a reliable instrument. In the whole sample, Cronbach's *α* coefficient was 0.93. It reached the same value in the middle and late adulthood age groups, whereas in the early adulthood group it was 0.91. For the individual SOC48-PL scales, Cronbach's alpha calculated for the whole sample reached values between 0.81 and 0.86, while calculated separately for the age groups, it reached values between 0.76 and 0.87. Reliability coefficients, including Cronbach’s *α,* Greatest Lower Bound (GLB), McDonald’s *ω*, and Guttman’s *λ6*, calculated for the whole sample and for particular age groups are given in Additional file 3: Table S1. Item-scale correlation analysis showed mostly moderate to high correlations (Additional file 4: Table S2), depending on the adopted interpretation [67, 73]. The absolute stability of SOC48-PL was assessed for a 1-month interval between the test and retest (± 3 days), in the late adulthood subgroup selected from the whole sample (n = 182, 40% men, age: 60–85 years, *M* = 67.47, *SD* = 6.43; years of education: *M* = 12.39, *SD* = 4.31). Correlations between the results obtained during the first test and retest were statistically significant (*p* < 0.001) and strong: Pearson’s correlations coefficients were 0.88 for the total score of SOC48-PL, and for scales: 0.83 (ES), 0.77 (LS), 0.88 (O), 0.81 (C). All test–retest correlations for individual items of the questionnaire were positive and significant at *p* < 0.001 and at the *q* < 0.001 when applying FDR correction for multiple comparisons. (Additional file 5: Table S3).

### Comparison of early, middle and late adulthood age groups in terms of SOC strategies, well-being and impulsivity

Comparison of the SOC strategies use in the age groups using one-way MANOVA test revealed statistically significant differences: *Wilks' Lambda* = 0.96, *p* = 0.001, but the effect size was very small: *partial η*^*2*^ = 0.02. Univariate ANOVA tests showed significant age-related differences only for the Loss-based selection scale: *F* = 3.01; *p* = 0.049, and the Optimization scale: *F* = 3.99; *p* = 0.02. Whereas according to the Bonferroni tests for multiple comparisons only the Optimization scale scores were found to be significantly lower in the late adulthood group compared to the middle adulthood group (*p* = 0.03). The descriptive statistics addressed by these comparisons are provided in Table 2. Moreover, according to the results of correlation analysis using Pearson's r coefficient, in all age groups individual SOC strategies were found to be significantly and positive inter-correlated. The strongest correlation regardless of group was revealed between optimization and compensation (Table 3). Furthermore, in most of the correlated pairs of scales, the strongest correlations occurred in the middle adulthood group (Table 3).

**Table 3:** Correlation coefficients (Pearson’s r) between measured variables in age groups of early, middle and late adulthood

SOC48-PL	SOC48-PL			SDHS	S UPPS-P				
	LS	O	C		NU	PU	LoPrem	LoPers	SS
Early adulthood									
ES	0.45***	0.53***	0.33***	0.12	− 0.27***	− 0.34***	− 0.36***	− 0.35***	− 0.01
LS		0.53***	0.52***	0.15	− 0.12	− 0.18*	− 0.29***	− 0.26***	− 0.18*
O			0.67***	0.23**	− 0.36***	− 0.37***	− 0.38***	− 0.40***	− 0.13
C				0.22**	− 0.12	− 0.13	− 0.26**	− 0.24***	− 0.18*
Middle adulthood									
ES	0.61***	0.58***	0.51***	0.16	− 0.10	− 0.21*	− 0.33***	− 0.38***	0.01
LS		0.56***	0.48***	0.06	0.04	− 0.02	− 0.35***	− 0.35***	0.04
O			0.75***	0.29***	− 0.15	− 0.18*	− 0.43***	− 0.47***	0.05
C				0.17	− 0.02	− 0.09	− 0.34***	− 0.35***	0.06
Late adulthood									
ES	0.48***	0.51***	0.37***	0.22**	− 0.12	− 0.12	− 0.34***	− 0.36***	0.01
LS		0.54***	0.51***	0.17*	− 0.10	− 0.13	0.26***	− 0.23**	0.001
O			0.66***	0.29***	− 0.13	− 0.13	− 0.36***	− 0.35***	0.07
C				0.20**	− 0.07	− 0.04	− 0.26***	− 0.15	0.08

*SOC48-PL* the SOC48-PL questionnaire, *ES* Elective selection, *LS* Loss-based selection, *O* Optimization, *C* Compensation, *SDHS* The Short Depression-Happiness Scale, *S UPPS-P* The Short UPPS-P Impulsivity Scale, *NU* Negative Urgency, *PU* Positive Urgency, *LoPrem* Lack of Premeditation, *LoPers* Lack of Perseverance, *SS* Sensation Seeking

Significance after applying the FDR correction for multiple comparisons: ***q < .001; **q < .01; *q < .05

As regards well-being, according to ANOVA, the SDHS scores obtained in the three age groups were not significantly different: *F* = 1.52; *p* = 0.23. Descriptive statistics for these scores are presented in Table 2.

In turn, MANOVA conducted for the impulsivity dimensions revealed the significant age-related differences: *Wilks' Lambda* = 8.15, *p* < 0.001; *partial η*^2^ = 0.07. Univariate ANOVA tests indicated that these significant differences concern the following S UPPS-P scales: Positive urgency (*F* = 5.97; *p* = 0.003), Lack of premeditation (*F* = 4.86; *p* = 0.008) and Sensation seeking (*F* = 24.65; *p* < 0.001). Based on the Bonferroni tests for multiple comparisons, it was found that positive urgency was significantly lower among older adults when compared to young adults (*p* = 0.008) and middle-aged adults (*p* = 0.014), whereas lack of premeditation was significantly lower in older adults in comparison to young adults (*p* = 0.007). Scores on the Lack of premeditation scale in the middle adulthood group took an intermediate level and were not significantly different from both other groups. Finally, sensation seeking in the early adulthood group was found to be significantly higher than in middle adulthood (*p* < 0.001) and late adulthood (*p* < 0.001). Descriptive statistics for impulsivity and its dimensions in the three age groups are shown in Table 2.

### Relationship of the SOC strategies use with well-being and impulsivity in three age groups

Overall, as indicated by the correlation analysis, the use of SOC strategies is positively related to well-being and negatively to impulsivity (Table 3).

The SOC strategies taken together were found to be most strongly associated with well-being in late adulthood, where scores of all SOC48-PL scales significantly correlate with SDHS scale (O scale: q < 0.001; ES and C scales: q < 0.01, and LS scale: q < 0.05). In contrast, in the middle adulthood group, the association of well-being with SOC strategies is the weakest. In this group significant correlation with SDHS score was noted only for optimization (O scale: q < 0.001). Moreover, the positive association of the SOC strategies use with well-being is visible especially for the optimization strategy—in all age groups the correlation coefficients of these variables were found to be statistically significant. Although it is more pronounced in late and middle adulthood, where the value of the correlation coefficient of the SDHS score with the O scale is clearly higher than the correlation with the other SOC48-PL scales, while among young adults SDHS scale correlates at similar levels two SOC48-PL scales: O scale and C scale.

Across all age groups, two dimensions of impulsivity correlated most strongly (and negatively) with the use of SOC strategies: lack of premeditation (LoPrem scale) and lack of perseverance (LoPers scale). At the same time, the strongest negative correlations with these two dimensions of impulsivity were shown for the optimization strategy. The scores of the O scale of the SOC48-PL questionnaire correlated significantly at the q < 0.01 level with the LoPrem and LoPers scales in all age groups, and the values of the correlation coefficients were higher than that of the other SOC 48-PL scales (except the late adulthood group, where the correlation coefficient value of the LoPers scale with the ES scale slightly exceeded this with the O scale). If correlation coefficients obtained across age groups are compared, the highest values were found in the middle adulthood group (from − 0.33 to − 0.47, all statistically significant), whereas slightly lower in groups of early adulthood (from − 0.24 to − 0.40, all statistically significant), and late adulthood (from − 0.15 to − 0.36, where the lowest correlation coefficient—for C scale—was not statistically significant).

Regarding the association of SOC strategies with urgency, statistically significant negative correlations were almost exclusively revealed in the group of young adults. In this group, negative and positive urgency (NU scale and PU scale) negatively correlated with optimization (O scale) and elective selection (ES scale) at q < 0.001. Moreover, positive urgency (PU scale) correlated negatively with loss-based selection strategy (LS scale) at q < 0.05. Among middle-aged and older adults, correlations coefficients with urgency were clearly lower and mostly not statistically significant. Only in the middle adulthood group negative and significant at q < 0.05 was correlation noted between positive urgency (PU scale) and two SOC strategies: optimisation and elective selection (O and ES scales). Sensation seeking (SS scale) was found to be significantly (q < 0.05) negatively associated only with loss-based selection and compensation strategies (LS and C scales) among young adults.

## Discussion

The study discussed in this article was the first one to measure SOC strategies in the Polish population, using a newly developed Polish version of the SOC Questionnaire. The aim of the study was, having assessed the reliability of the questionnaire, to compare the age groups of early, middle and late adulthood in their use of SOC strategies, and to investigate the relationship of the use of SOC strategies in these groups with well-being and impulsivity.

### Reliability of the Polish version of the SOC Questionnaire

The analysis of psychometric properties of SOC48-PL undertaken at the outset showed that the instrument was reliable in terms of its internal consistency and absolute stability. This applies both to the questionnaire as a whole and to individual scales. However, further research into the reliability and validity of the tool will improve its trustworthiness.

### Comparison of the SOC strategies use in early, middle and late adulthood

Regarding age-related differences in the use of the SOC strategies, when compared with the mean scores obtained on the individual SOC48-PL scales, supported by a significant MANOVA result, the pattern obtained in the present study is consistent with that observed by the authors of the original version of the questionnaire [3]. The highest scores on the scales measuring loss-based selection, optimization and compensation strategies were found in middle adulthood, which is to reflect the ‘developmental peak’ of these three strategies during this stage of life. In turn, the mean scores on the scale measuring elective selection increased with age. On the contrary, the assumption on a more intensive use of loss-related strategies among older as opposed to younger adults [26, 28, 74] was not confirmed by the obtained results. However, first and foremost, the majority of differences in mean scores between age groups were, according to one-way ANOVAs, statistically insignificant. Moreover, the inclusion of post-hoc tests for ANOVA basically revealed only a significantly higher tendency to optimization in middle adulthood compared to late adulthood. Taking this into account, it is reasonable to conclude that developmental differences in the use of SOC strategies are negligible. In addition, the small differences between the age groups are accompanied by a considerable variance in results within these groups. This raises the conclusion that individual differences determine the use of SOC strategies to at least the same extent as developmental changes. While there has been limited number of studies aimed directly at comparing age groups in terms of their use of SOC strategies, it is important to recognise that the results of the research presented here do not confirm the existence of age differences indicated by previous findings. The reason for this is not clear. This could be related to cross-cultural differences and the specificity of the Polish population. In addition, the use of SOC strategies across age groups may change across generations and formerly more pronounced differences may currently be decreasing. Finally, it is possible that despite small quantitative differences between the age groups, these groups differ in qualitative ways in their use of SOC strategies. However, all these possibilities are hypothetical and would require future research. Going further, regardless of age group, individual SOC strategies proved to be interrelated. At the same time, the strongest associations between strategies emerged in the middle adulthood group. This applies especially to the relationship between strategies of achieving goals: optimisation and compensation—strongly linked also in other age groups, but also to the relationship between selection strategies (elective and loss-based)—which, in turn, correlated clearly as being weaker in the other age groups. This finding is coherent with the assumption of the highest consistency of SOC strategies in middle adulthood [3].

### Relationship between the SOC strategies use and well-being

The present study confirmed the positive relationship between the use of SOC strategies and well-being. This association has already been demonstrated in many studies, as indicated in the introduction. The strongest link observed in the group of older adults confirms the special role of SOC strategies for well-being at this stage of development, postulated in the SOC Model [1, 21] and indicated in previous studies [e.g., 29, 35, 38]. Well-being was found to be associated particularly strongly with goal-oriented strategies. In the late and middle adulthood groups, this mainly concerned optimisation, which is the strategy employed when resources are sufficiently available. In contrast, among young adults, the association of well-being with optimisation was similarly strong to that with compensation, which is used when certain resources are not available. This result can be attributed to a higher probability of only temporary unavailability of resources in early adulthood, with the perspective of future expansion of resources, and permanent unavailability of resources in older groups, due to the subsequent decline in resources with age. As a result, young adults may use optimization and compensation in a more dynamic and alternate way, and consequently feel less of an ‘advantage’ in being able to use optimization instead of compensation. However, it is worth noting that the strength of the correlation between the use of SOC strategies and well-being in the Polish sample was relatively lower when compared to that reported in other studies. It is not clear whether this is due to the specificity of the study population, related to its socio-cultural context, or to the specificity of the well-being measurement instrument employed in the study.

### Relationship between the SOC strategies use and impulsivity

In the present study, the relationship between the use of SOC strategies and impulsivity, assumed from theoretical analysis, was examined for the first time. Negative correlations of SOC strategies with impulsivity can be interpreted in terms of opposition between intentional self-regulation and impulsivity. Therefore, it was expected that such a negative relationship would primarily concern two dimensions of impulsivity, i.e., lack of premeditation and lack of perseverance. The results obtained confirmed that in all age groups these two impulsivity facets are most strongly negatively associated with SOC strategies. On the basis of theoretical deliberations, it was hypothesised that lack of premeditation would correlate negatively with all SOC strategies, whereas lack of perseverance would correlate with those related to goal attainment, namely optimization and compensation. However, the results revealed that lack of persistence is also negatively associated with SOC strategies related to planning: elective selection and loss-based selection. This may be attributable to the already mentioned functioning of the SOC strategies on an ‘orchestration’ basis, i.e., as remaining in mutual interaction and cooperation [2, 22]. The strongest association of lack of premeditation and lack of perseverance with SOC strategies was revealed in middle adulthood, what may be attributable to the ‘developmental peak’ of the SOC strategies in this developmental period [3]. In turn, comparing the SOC strategies, the strongest negative relationship with lack of premeditation and lack of perseverance was observed for optimization. This result can be explained by the fact that optimization is strategy reflected in how effectively the person acts while pursuing goals, in particular by focussing attention on the goal, choosing the right moment of action, making proper allocation of resources that facilitate the achievement of the goal, undertaking effort, practicing skills, and being persistent [3, 22]. These behavioural traits directly relate to premeditation and perseverance, the deficits of which are part of impulsivity. In contrast, and surprisingly, for compensation in the late adulthood group, the assumed negative link with lack of perseverance proved to be statistically insignificant, although it was significant in the other age groups. It is difficult to identify the source of such a result. It may have the same origins as another result of the present study, which was found to be inconsistent with previous findings, namely the lack of advantage of persons in late adulthood over young adults in using compensation as loss-related strategy. This can suggest the existence of a particular specificity of the use of compensation strategy in the Polish population. In this regard, the use of compensation strategy among Poles, especially in late adulthood, should be looked at more closely and take into account a qualitative approach and the context of Polish culture. Going further, negative link between SOC strategies and urgency occurred mainly in the youngest group, that may indicate a changing role of impulsivity dimensions in the course of development. In young adults, the tendency to behave rashly under negative emotions was not stronger than in other age groups, and under positive emotions it was stronger only compared to older adults. In spite of it, in early adulthood, urgency and SOC strategies were more negatively interrelated than in middle and late adulthood. This may mean that in early adulthood urgency plays a more important role in an individual's functioning, being more strongly associated with its other facets. Sensation seeking proved to be not related to SOC strategies use, except for early adulthood group. This corresponds with the quite frequent treatment of sensation seeking as distinguished from other forms of impulsivity and also associated with positive phenomena [66, 75, 76]. However, among young adults, sensation seeking was negatively associated with loss-based selection and compensation strategies. Both of these strategies are triggered in response to resource constraints. Their involvement, in a sense, indicates acceptance of this situation and an attempt to adapt to it. It seems that in early adulthood the tendency to seek out novel and thrilling experiences remains in stronger opposition to the acceptance of the limitations of one's capacities than in later periods of development.

### Limitations

The presented study is not free from limitations. We used the snowball sampling procedure, while random sampling would be best to create a representative sample. In addition, the three age groups differed in terms of size. They were also not perfectly adjusted in terms of sex and education level, and women and better educated people were overrepresented. In addition, only some study participants representing just one age group (late adulthood) were involved in testing the test–retest reliability of the instrument. In evaluating differences between early, middle and late adulthood, the cross-sectional comparisons were used. Longitudinal studies provide better information on developmental changes. However, there is a general lack of longitudinal research on changes in the use of SOC strategies over the life span. Moreover, as the Polish version of the SOC strategies measurement tool has only recently been developed, only now can longitudinal studies among Poles be initiated. It is also important to be aware that the study was conducted on a correlational model, so causal relationships between the use of SOC strategies and well-being and impulsivity cannot be confidently inferred.

## Practical implications

The baseline for the present study was the development of a Polish version of a questionnaire to measure SOC strategies: SOC48-PL. It can be considered useful and applied in research on the SOC model in the Polish population, as well as in cross-cultural studies that include this population and aim at comparing it with others. Moreover, this questionnaire can be used in psychological (as well as medical and nursing) practice to evaluate individual Polish speaker’s strategies for manage and coping with changing life circumstances, and afterwards to support the SOC strategies use that promote self-regulation and living a good life in spite of difficulties, losses and disabilities. Furthermore, the positive association of SOC strategies with well-being, also demonstrated in the Polish sample, combined with previous reports of successful outcomes of SOC strategy use-training [19, 20], confirms the value of designing this type of intervention and suggests the rationale for introducing it in the Polish population. As Polish society belongs to the ageing societies [77] and the SOC model was originally developed as a theory for successful ageing, interventions based on the SOC model seem to be particularly needed among persons in late adulthood, although they should not be limited only to this age group. Similarly, the negative association of SOC strategies with impulsivity found in this study, primarily with lack of premeditation and lack of perseverance, is an indication suggesting the potential value of interventions based on teaching the use of SOC strategies as ameliorating or offsetting the negative consequences of impulsivity.

### Recommendations for further research

In the present study, a newly developed Polish version of the SOC questionnaire was used. The analyses conducted indicate that the tool is reliable. However, further research to confirm the reliability and validity of the tool would be desirable. First of all, its validity in the Polish population should be verified by linking SOC strategies with a wide and diverse set of psychological variables, including personality variables for which an association with SOC strategies has been demonstrated in previous studies, additionally measured by different instruments, not only questionnaire-based, to avoid the common method bias [78]. Cross-cultural comparisons are also an interesting, if not essential, direction for further research. Especially when taking into account some discrepancies in the results of the study in the Polish sample in relation to reports from earlier studies. However, conducting such comparisons would require prior demonstration of measurement equivalence of respective versions (used in compared cultures) of questionnaires for measuring SOC strategies. Furthermore, in view of the negligible age-related differences revealed in the present study, which was cross-sectional in nature, and the general scarcity of long-term longitudinal studies on the use of SOC strategies, undertaking a longitudinal study in this area would be of great value. Cross-sectional data are confounded by the cohort effect. Moreover, they do not allow one to determine the causal or temporal nature of the association between the use of SOC strategies and the other psychological variables. Longitudinal studies would allow handling these limitations. Even better would be a cross-sequential design allowing not only the control of the cohort effect but also its evaluation. The reason is, that asking about intergenerational differences in the use of SOC strategies is no less interesting than asking about changes in their use with age. Finally, in the light of the results obtained in this study and of some of their discrepancies with the results of previous studies, it seems reasonable to combine quantitative research with qualitative research. The latter could provide better insights into how SOC strategies are used in action and into the nature of the relationships between individual strategies. This in turn could allow for a better explanation of the quantitative results obtained.

## Conclusions

The present study used the newly created Polish version of the SOC Questionnaire to measure and compare the use of the SOC strategies in the early, middle and late adulthood age groups, and to test the relationship of these strategies’ use with well-being and impulsivity. The results obtained allow us to consider the Polish version of the SOC questionnaire as reliable. In the light of the study’s findings, age-related differences in the use of SOC strategies are rather slight, and overlapped with substantial individual differences. The study provides some indicators of the existence of the SOC strategies’ ‘developmental peak’ in middle adulthood, but the indicators do not allow for definitive conclusions. As was predicted and in line with the results of previous studies, the use of SOC strategies was found to be positively associated with well-being in all age groups, especially in older adults [3, 7, 17, 29, 35, 38]. The relationship between the SOC strategy use and impulsivity, hypothesised from the theoretical analysis and investigated for the first time in the present study, proved to be significantly negative in all age groups, particularly with two of its dimensions: lack of premeditation and lack of perseverance. Associations of SOC strategies with urgency were noticeably weaker, while with sensation seeking hardly ever occurred. The negative association of SOC strategy use with impulsivity may suggest that for impulsive individuals, spontaneous use of these life-management strategies may be difficult and less likely than those with low impulsivity. The present study has some practical implications. The newly developed SOC48-PL questionnaire may be used to measure SOC strategies in subsequent studies in the Polish population or in cross-cultural studies including the aforementioned population. It may also find application in psychological practice when an evaluation of an individual's coping strategies with the changing context of life, including losses and limitations, is needed. Furthermore, the confirmed association of SOC strategies with well-being and their newly identified association with impulsivity may provide a starting rationale for designing practical interventions. These interventions could teach the use of SOC strategies in the context of providing well-being, such as among older adults, and in the context of reducing the impact of impulsivity on managing the course of one's life and development. In further research, it is worthwhile to verify more extensively the reliability and validity of the SOC48-PL, so that this questionnaire can also be used in cross-cultural studies. Longitudinal studies of the evolution of the use of the SOC strategies over the life course would also be an important challenge. In turn, combining quantitative and qualitative research would provide greater insight into the ways in which SOC strategies are used in practice and produce explanations for unexpected quantitative results.

## Supplementary Information

**Additional file 1.**: SOC48-PL. SOC48-PL questionnaire in the graphic version used in the study, in English

**Additional file 2. Fig. S1 **: The procedure of the SOC48-PL questionnaire development. Graphical illustration of the procedure of development of the Polish version of the SOC-Questionnaire, including translation and back-translation from the two original language versions (English and German) and evaluation by competent judges.

**Additional file 3. Table S1 **: Reliability statistics of the SOC48-PL and their individual scales. Reliability coefficients (McDonald's *ω*, Greatest lower bound, Guttman's *λ6*, and Cronbach's *α* with 95% Confidence Interval) of the Polish version of the SOC questionnaire and its individual scales in the whole study sample and age groups of early, middle and late adulthood.

**Additional file 4. Table S2**: Statistics for items of individual scales based on reliability analysis. Item-scale correlation coefficients and Cronbach's *α*when item is deleted for each item of particular SOC48-PL questionnaire scales

**Additional file 5. Table S3**: Pearson's correlations coefficients between the results obtained during the first test and retest in particular items of the SOC48-PL questionnaire. Correlation coefficients (Pearson's *r*) for individual SOC48-PL questionnaire items obtained in the test-retest reliability evaluation procedure

## Data Availability

The datasets generated and analysed during the current study are available in Mendeley Data repository, http://dx.doi.org/10.17632/kz85yr74g7.1

## Abbreviations

C: Compensation
ES: Elective selection
FDR: False Discovery Rate correction for multiple comparisons
GLB: Greatest lower bound
LoPers: Lack of Perseverance
LoPrem: Lack of Premeditation
LS: Loss-based selection
NU: Negative urgency
O: Optimization
PU: Positive Urgency
PYD: Positive youth development
S UPPS-P: The Short UPPS-P Impulsivity Scale
SDHS: The Short Depression-Happiness Scale
SOC: Selection, optimization, and compensation
SOC48-PL: The Selection, Optimization, and Compensation Questionnaire, Polish version
SS: Sensation Seeking
