# Supplementary material for: Selection, optimization and compensation strategies and their relationship with well-being and impulsivity in early, middle and late adulthood in a Polish sample
Source: BMC Psychol. 2021 Sep 16;9:144. doi: 10.1186/s40359-021-00650-2 (PMC8447622; doi:10.1186/s40359-021-00650-2)
Supplement: Supplementary file 1 — Additional file 1.: SOC48-PL. SOC48-PL questionnaire in the graphic version used in the study, in English [file 40359_2021_650_MOESM1_ESM.docx]

**Selection, optimization and compensation strategies and their relationship with well-being and impulsivity in early, middle and late adulthood in a Polish sample**

Ludmiła Zając-Lamparska^1^

^1^ Faculty of Psychology, Kazimierz Wielki University in Bydgoszcz, Poland

**Author Note**

Ludmiła Zając-Lamparska [
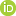
](https://orcid.org/0000-0003-4618-547X) https://orcid.org/0000-0003-4618-547X

Correspondence concerning this article should be addressed to Ludmiła Zając-Lamparska, Faculty of Psychology, Kazimierz Wielki University, ul. Staffa 1, 85-867 Bydgoszcz, Poland. Email: [lzajac@ukw.edu.pl](mailto:lzajac@ukw.edu.pl)

**SOC48-PL**

(on the basis of *SOC-Questionnaire* by P. B. Baltes, M. M. Baltes A. M. Freund i F. Lang)

**Instruction**

**We are very interested in learning about how you decide which things in life are important for you and how you go about accomplishing what you want in life.**

**In the following, we present examples of two different ways people might behave. Imagine there are two people talking about what they would do in a particular situation. We would like you to decide which person is most similar to you - in other words, which one behaves most like the way you probably would.**

**Now, think about your life overall, including how things are going, think about your goals – that is, both things that you want to improve and things that you are satisfied with and want to maintain.**

In answering, always indicate first whether person A or person B is more like you.

For example, if you think that person A is more like you, tick the box with the letter A, as in the example below:

| A | B |
| --- | --- |

Then state more precisely to what extent your behaviour would be similar to the described behaviour of persons A and B. Tick the box: the closer you get to A, the more similar your behaviour would be to that of person A, and the closer you get to B, the more similar your behaviour would be to that of person B.

For example, if you think that your behaviour would be exactly like that of person B, tick the box closest to B, as in the example below:

| A |  |  |  |  |  |  | B |
| --- | --- | --- | --- | --- | --- | --- | --- |

And if, for example, you think that your behaviour would be just a little bit more like that of person A than person B, tick the one in the middle which is slightly closer to A than to B, as in the example below:

| A |  |  |  |  |  |  | B |
| --- | --- | --- | --- | --- | --- | --- | --- |

**ES Scale**

ES1

| Person A | Person B |
| --- | --- |
| I concentrate all my energy on few things. | I divide my energy among many things. |

| A | B |
| --- | --- |

| A |  |  |  |  |  |  | B |
| --- | --- | --- | --- | --- | --- | --- | --- |

ES2

| Person A | Person B |
| --- | --- |
| I always focus on the one most important goal at a given time. | I am always working on several goals at once. |

| A | B |
| --- | --- |

| A |  |  |  |  |  |  | B |
| --- | --- | --- | --- | --- | --- | --- | --- |

ES3

| Person A | Person B |
| --- | --- |
| When I think about what I want in life, I commit myself to one or two important goals. | Even when I really consider what I want in life, I wait and see what happens, instead of committing myself to just one or two particular goals. |

| A | B |
| --- | --- |

| A |  |  |  |  |  |  | B |
| --- | --- | --- | --- | --- | --- | --- | --- |

ES4

| Person A | Person B |
| --- | --- |
| To achieve an important goal, I am willing to postpone other goals. | Just to achieve an important goal, I am not willing to postpone other goals. |

| A | B |
| --- | --- |

| A |  |  |  |  |  |  | B |
| --- | --- | --- | --- | --- | --- | --- | --- |

ES5

| Person A | Person B |
| --- | --- |
| I always pursue goals one after the other. | I always pursue many goals at once, so that I easily get bogged down. |

| A | B |
| --- | --- |

| A |  |  |  |  |  |  | B |
| --- | --- | --- | --- | --- | --- | --- | --- |

ES6

| Person A | Person B |
| --- | --- |
| I know exactly what I want and what I don't want. | I often only know what I want as the result of a situation. |

| A | B |
| --- | --- |

| A |  |  |  |  |  |  | B |  |
| --- | --- | --- | --- | --- | --- | --- | --- | --- |

ES7

| Person A | Person B |
| --- | --- |
| When I decide upon a goal, I stick to it. | I can change a goal again at any time. |

| A | B |
| --- | --- |

| A |  |  |  |  |  |  | B |
| --- | --- | --- | --- | --- | --- | --- | --- |

ES8

| Person A | Person B |
| --- | --- |
| I always direct my attention to my most important goal. | I always approach several goals at once. |

| A | B |
| --- | --- |

| A |  |  |  |  |  |  | B |
| --- | --- | --- | --- | --- | --- | --- | --- |

ES9

| Person A | Person B |
| --- | --- |
| I make important life decisions. | I don't like to commit myself specific life decisions. |

| A | B |
| --- | --- |

| A |  |  |  |  |  |  | B |
| --- | --- | --- | --- | --- | --- | --- | --- |

ES10

| Person A | Person B |
| --- | --- |
| I consider exactly what is important for me. | I take things as they come and carry on from there. |

| A | B |
| --- | --- |

| A |  |  |  |  |  |  | B |
| --- | --- | --- | --- | --- | --- | --- | --- |

ES11

| Person A | Person B |
| --- | --- |
| I don't have many goals in life that are equally important to me. | I have many goals in life that are equally important to me. |

| A | B |
| --- | --- |

| A |  |  |  |  |  |  | B |
| --- | --- | --- | --- | --- | --- | --- | --- |

ES12

| Person A | Person B |
| --- | --- |
| I have set my goals clearly and stick to them. | I often adapt my goals to small changes. |

| A | B |
| --- | --- |

| A |  |  |  |  |  |  | B |
| --- | --- | --- | --- | --- | --- | --- | --- |

**LS Scale**

LS1

| Person A | Person B |
| --- | --- |
| When things don't go as well as before, I choose one or two important goals. | When things don't go as well as before, I still try to keep all my goals. |

| A | B |
| --- | --- |

| A |  |  |  |  |  |  | B |
| --- | --- | --- | --- | --- | --- | --- | --- |

LS2

| Person A | Person B |
| --- | --- |
| When I can't do something important the way I did before, I look for a new goal. | When I can't do something important the way I did before, I distribute my time and energy among many other things. |

| A | B |
| --- | --- |

| A |  |  |  |  |  |  | B |
| --- | --- | --- | --- | --- | --- | --- | --- |

LS3

| Person A | Person B |
| --- | --- |
| When I can't do something as well as I used to, I think about what exactly is important to me. | When I can't do something as well as I used to, I wait and see what comes. |

| A | B |
| --- | --- |

| A |  |  |  |  |  |  | B |
| --- | --- | --- | --- | --- | --- | --- | --- |

LS4

| Person A | Person B |
| --- | --- |
| If l can't do something as well as before, I concentrate only on essentials | Even if l can't do something as well as before, I pursue all my goals. |

| A | B |
| --- | --- |

| A |  |  |  |  |  |  | B |
| --- | --- | --- | --- | --- | --- | --- | --- |

LS5

| Person A | Person B |
| --- | --- |
| When I can't carry on as I used to, I direct my attention to my most important goal. | When I can't carry on as I used to, I direct my attention, like usual, to all my goals. |

| A | B |
| --- | --- |

| A |  |  |  |  |  |  | B |
| --- | --- | --- | --- | --- | --- | --- | --- |

LS6

| Person A | Person B |
| --- | --- |
| When something becomes increasingly difficult for me, I consider which goals I could achieve under the circumstances. | When something becomes increasingly difficult for me, I accept it. |

| A | B |
| --- | --- |

| A |  |  |  |  |  |  | B |  |
| --- | --- | --- | --- | --- | --- | --- | --- | --- |

LS7

| Person A | Person B |
| --- | --- |
| When things don't work so well, I pursue my most important goal first. | When things don't go so well, I leave it at that. |

| A | B |
| --- | --- |

| A |  |  |  |  |  |  | B |
| --- | --- | --- | --- | --- | --- | --- | --- |

LS8

| Person A | Person B |
| --- | --- |
| When something requires more and more effort, I think about what exactly I really want. | When something requires more and more effort, I don't worry about it. |

| A | B |
| --- | --- |

| A |  |  |  |  |  |  | B |
| --- | --- | --- | --- | --- | --- | --- | --- |

LS9

| Person A | Person B |
| --- | --- |
| When things don't go as well as before, I drop some goals to concentrate on the more important ones. | When things don't go as well as before, I wait for better times. |

| A | B |
| --- | --- |

| A |  |  |  |  |  |  | B |
| --- | --- | --- | --- | --- | --- | --- | --- |

LS10

| Person A | Person B |
| --- | --- |
| When I am not able to achieve something any more, I direct my efforts at what is still possible. | When I am not able to achieve something any more, I trust that the situation will improve by itself. |

| A | B |
| --- | --- |

| A |  |  |  |  |  |  | B |
| --- | --- | --- | --- | --- | --- | --- | --- |

LS11

| Person A | Person B |
| --- | --- |
| When things don't go as well as before, I think about what, exactly, is really important to me. | When things don't go as well as before, I leave it at that. |

| A | B |
| --- | --- |

| A |  |  |  |  |  |  | B |
| --- | --- | --- | --- | --- | --- | --- | --- |

LS12

| Person A | Person B |
| --- | --- |
| When I can no longer do something in my usual way, I think about what, exactly, I am able to do under the circumstances. | When I can no longer do something in my usual way, I don't think long about it. |

| A | B |
| --- | --- |

| A |  |  |  |  |  |  | B |
| --- | --- | --- | --- | --- | --- | --- | --- |

**O Scale**

O1

| Person A | Person B |
| --- | --- |
| I keep trying as many different possibilities as are necessary to succeed at my goal. | When I do not succeed right away at what I want to do, I don't try other possibilities for very long. |

| A | B |
| --- | --- |

| A |  |  |  |  |  |  | B |
| --- | --- | --- | --- | --- | --- | --- | --- |

O2

| Person A | Person B |
| --- | --- |
| I make every effort to achieve a given goal. | I prefer to wait for a while and see if things will work out by themselves. |

| A | B |
| --- | --- |

| A |  |  |  |  |  |  | B |
| --- | --- | --- | --- | --- | --- | --- | --- |

O3

| Person A | Person B |
| --- | --- |
| - 1. If something matters to me, I devote myself fully and completely to it. | Even if when something matters to me, I still have a hard time devoting myself fully and completely to it. |

| A | B |
| --- | --- |

| A |  |  |  |  |  |  | B |
| --- | --- | --- | --- | --- | --- | --- | --- |

O4

| Person A | Person B |
| --- | --- |
| I keep trying until I succeed at a goal. | I don't keep trying very long, when I don't succeed right away at a goal. |

| A | B |
| --- | --- |

| A |  |  |  |  |  |  | B |
| --- | --- | --- | --- | --- | --- | --- | --- |

O5

| Person A | Person B |
| --- | --- |
| I do a lot myself to realize my goals. | I trust that my goals someday come true. |

| A | B |
| --- | --- |

| A |  |  |  |  |  |  | B |
| --- | --- | --- | --- | --- | --- | --- | --- |

O6

| Person A | Person B |
| --- | --- |
| When I choose a goal, I am also willing to invest much effort in it. | I usually choose a goal that I can achieve without much effort. |

| A | B |
| --- | --- |

| A |  |  |  |  |  |  | B |  |
| --- | --- | --- | --- | --- | --- | --- | --- | --- |

O7

| Person A | Person B |
| --- | --- |
| When I want to achieve something difficult, I wait for the right moment and the best opportunity. | When I want to achieve something difficult, I don’t want to wait long for the very best opportunity. |

| A | B |
| --- | --- |

| A |  |  |  |  |  |  | B |
| --- | --- | --- | --- | --- | --- | --- | --- |

O8

| Person A | Person B |
| --- | --- |
| When I have started something that is important to me, but has little chance at success, I make a particular effort. | When I start something that is important to me but has little chance at success, I usually stop trying. |

| A | B |
| --- | --- |

| A |  |  |  |  |  |  | B |
| --- | --- | --- | --- | --- | --- | --- | --- |

O9

| Person A | Person B |
| --- | --- |
| When I want to get ahead, I take a successful persons as a model. | When I want to get ahead, only I myself know the best way to do it. |

| A | B |
| --- | --- |

| A |  |  |  |  |  |  | B |
| --- | --- | --- | --- | --- | --- | --- | --- |

O10

| Person A | Person B |
| --- | --- |
| I think about exactly how I can best realize my plans. | I don't think long about how to realize my plans, I just try it. |

| A | B |
| --- | --- |

| A |  |  |  |  |  |  | B |
| --- | --- | --- | --- | --- | --- | --- | --- |

O11

| Person A | Person B |
| --- | --- |
| When something is important to me, I don't let setbacks discourage me. | Setbacks show me that I should turn to something else. |

| A | B |
| --- | --- |

| A |  |  |  |  |  |  | B |
| --- | --- | --- | --- | --- | --- | --- | --- |

O12

| Person A | Person B |
| --- | --- |
| When realizing my plans, I think carefully about the when and how to do it. | When realizing my plans, I typically start right away. |

| A | B |
| --- | --- |

| A |  |  |  |  |  |  | B |
| --- | --- | --- | --- | --- | --- | --- | --- |

**C Scale**

C1

| Person A | Person B |
| --- | --- |
| When things don't go as well as they used to, I keep trying other ways until I can achieve the same result I used to. | When things don't go as well as they used to, I accept it. |

| A | B |
| --- | --- |

| A |  |  |  |  |  |  | B |
| --- | --- | --- | --- | --- | --- | --- | --- |

C2

| Person A | Person B |
| --- | --- |
| When something in my life isn't working as well as it used to, I ask others for help or advice. | When something in my life isn't working as well as it used to, I decide what to do about it myself, without involving other people. |

| A | B |
| --- | --- |

| A |  |  |  |  |  |  | B |
| --- | --- | --- | --- | --- | --- | --- | --- |

C3

| Person A | Person B |
| --- | --- |
| When it becomes harder for me to get the same results, I keep trying harder until I can do it as well as before. | When it becomes harder for me to get the same results as I used to, it is time to let go of that expectation. |

| A | B |
| --- | --- |

| A |  |  |  |  |  |  | B |
| --- | --- | --- | --- | --- | --- | --- | --- |

C4

| Person A | Person B |
| --- | --- |
| For important things, I pay attention to whether I need to devote more time or effort. | Even if something is important to me, it can happen that I don't invest the necessary time or effort. |

| A | B |
| --- | --- |

| A |  |  |  |  |  |  | B |
| --- | --- | --- | --- | --- | --- | --- | --- |

C5

| Person A | Person B |
| --- | --- |
| In particularly difficult life situations, I try to get help from doctors, counselors or other experts. | In particularly difficult life situations, I try to manage by myself |

| A | B |
| --- | --- |

| A |  |  |  |  |  |  | B |
| --- | --- | --- | --- | --- | --- | --- | --- |

C6

| Person A | Person B |
| --- | --- |
| When things aren't going so well, I accept help from others. | Even in difficult situations, I don't burden others. |

| A | B |
| --- | --- |

| A |  |  |  |  |  |  | B |  |
| --- | --- | --- | --- | --- | --- | --- | --- | --- |

C7

| Person A | Person B |
| --- | --- |
| When things don't work the way they used to, I look for other ways to achieve them. | When things don't work the way they used to, I accept things the way they are. |

| A | B |
| --- | --- |

| A |  |  |  |  |  |  | B |
| --- | --- | --- | --- | --- | --- | --- | --- |

C8

| Person A | Person B |
| --- | --- |
| When I can't do something as well as before, then I find out about other ways and means to achieve it. | When I can't do something as well as before, then I accept it. |

| A | B |
| --- | --- |

| A |  |  |  |  |  |  | B |
| --- | --- | --- | --- | --- | --- | --- | --- |

C9

| Person A | Person B |
| --- | --- |
| When I can't do something as well as I used to, then I ask someone else to do it for me. | When I can't do something as well as I used to, I accept the change. |

| A | B |
| --- | --- |

| A |  |  |  |  |  |  | B |
| --- | --- | --- | --- | --- | --- | --- | --- |

C10

| Person A | Person B |
| --- | --- |
| When I am afraid of losing something that I've achieved, then I invest more time and effort in it. | Just to prevent losing what I've achieved, I'm not willing to invest more time and effort in it. |

| A | B |
| --- | --- |

| A |  |  |  |  |  |  | B |
| --- | --- | --- | --- | --- | --- | --- | --- |

C11

| Person A | Person B |
| --- | --- |
| When something doesn't work as well as usual, I look at how others do it. | When something doesn't work as well as usual, I don't spend much time thinking about it. |

| A | B |
| --- | --- |

| A |  |  |  |  |  |  | B |
| --- | --- | --- | --- | --- | --- | --- | --- |

C12

| Person A | Person B |
| --- | --- |
| When something does not work as well as before, I listen to advisory broadcasts and books as well | When something does not work as well as before, I am the one who knows what is best for me. |

| A | B |
| --- | --- |

| A |  |  |  |  |  |  | B |
| --- | --- | --- | --- | --- | --- | --- | --- |

Thank you for completing the questionnaire!
