# Supplementary material for: Selection, optimization and compensation strategies and their relationship with well-being and impulsivity in early, middle and late adulthood in a Polish sample
Source: BMC Psychol. 2021 Sep 16;9:144. doi: 10.1186/s40359-021-00650-2 (PMC8447622; doi:10.1186/s40359-021-00650-2)
Supplement: Supplementary file 2 — Additional file 2. Fig. S1 : The procedure of the SOC48-PL questionnaire development. Graphical illustration of the procedure of development of the Polish version of the SOC-Questionnaire, including translation and back-translation from the two original language versions (English and German) and evaluation by competent judges. [file 40359_2021_650_MOESM2_ESM.docx]

7

**Selection, optimization and compensation strategies and their relationship with well-being and impulsivity in early, middle and late adulthood in a Polish sample**

Ludmiła Zając-Lamparska^1^

^1^ Faculty of Psychology, Kazimierz Wielki University in Bydgoszcz, Poland

**Author Note**

Ludmiła Zając-Lamparska [
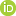
](https://orcid.org/0000-0003-4618-547X) https://orcid.org/0000-0003-4618-547X

Correspondence concerning this article should be addressed to Ludmiła Zając-Lamparska, Faculty of Psychology, Kazimierz Wielki University, ul. Staffa 1, 85-867 Bydgoszcz, Poland. Email: [lzajac@ukw.edu.pl](mailto:lzajac@ukw.edu.pl)

Figure 1. *The procedure of the SOC48-PL questionnaire development*

SOC-Questionnaire in English (48 items)

SOC-Questionnaire in Polish – version 1^d^

SOC-Questionnaire in German (48 items)

translation to Polish^a^

back-translation to English^c^

translation to Polish^b^

Evaluation and correction by five competent judges (psychologists)^e^

SOC-Questionnaire in Polish – version 2 (SOC48-PL)^f^

*Note:*

^a^ Translation by a professional translator, a Polish origin, a master's degree in English and psychology

^b^ Translation by the author of the article

^c^ Back-translation by a psychologist with a PhD degree obtained in the United States, a Polish of origin, currently academic active in the United States and Poland

^d^ Determination the wording of individual items of the questionnaire and the user manual in Polish by the author of the article, based on both translated versions, including back-translation into English

^e^ Polish psychologists with at least a doctoral degree

^f^ Development of the final version of the SOC48-PL by the author of the article, after considering the opinions and comments of competent judges
