# Supplementary material for: Selection, optimization and compensation strategies and their relationship with well-being and impulsivity in early, middle and late adulthood in a Polish sample
Source: BMC Psychol. 2021 Sep 16;9:144. doi: 10.1186/s40359-021-00650-2 (PMC8447622; doi:10.1186/s40359-021-00650-2)
Supplement: Supplementary file 3 — Additional file 3. Table S1 : Reliability statistics of the SOC48-PL and their individual scales. Reliability coefficients (McDonald's ω, Greatest lower bound, Guttman's λ6, and Cronbach's α with 95% Confidence Interval) of the Polish version of the SOC questionnaire and its individual scales in the whole study sample and age groups of early, middle and late adulthood. [file 40359_2021_650_MOESM3_ESM.docx]

**Selection, optimization and compensation strategies and their relationship with well-being and impulsivity in early, middle and late adulthood in a Polish sample**

Ludmiła Zając-Lamparska^1^

^1^ Faculty of Psychology, Kazimierz Wielki University in Bydgoszcz, Poland

**Author Note**

Ludmiła Zając-Lamparska [
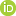
](https://orcid.org/0000-0003-4618-547X) https://orcid.org/0000-0003-4618-547X

Correspondence concerning this article should be addressed to Ludmiła Zając-Lamparska, Faculty of Psychology, Kazimierz Wielki University, ul. Staffa 1, 85-867 Bydgoszcz, Poland. Email: [lzajac@ukw.edu.pl](mailto:lzajac@ukw.edu.pl)

**Table 1**

*Reliability statistics of* *the* *SOC48-PL and their individual scales*

|  |  |  |  |  | 95% Confidence Interval | |
| --- | --- | --- | --- | --- | --- | --- |
|  | McDonald’s *ω* | GLB | Guttman’s *λ6* | Cronbach’s *α* | Lower | Upper |
| Whole sample | | | | | | |
| SOC | 0.93 | 0.93 | 0.95 | 0.93 | 0.92 | 0.93 |
| ES | 0.82 | 0.87 | 0.83 | 0.82 | 0.79 | 0.84 |
| LS | 0.81 | 0.88 | 0.82 | 0.81 | 0.79 | 0.83 |
| O | 0.87 | 0.91 | 0.87 | 0.86 | 0.84 | 0.87 |
| C | 0.83 | 0.89 | 0.86 | 0.83 | 0.81 | 0.85 |
| Early adulthood | | | | | | |
| SOC | 0.92 | 0.91 | 0.95 | 0.91 | 0.90 | 0.93 |
| ES | 0.79 | 0.87 | 0.80 | 0.78 | 0.73 | 0.84 |
| LS | 0.77 | 0.87 | 0.79 | 0.76 | 0.71 | 0.81 |
| O | 0.85 | 0.91 | 0.86 | 0.83 | 0.80 | 0.87 |
| C | 0.83 | 0.88 | 0.85 | 0.83 | 0.79 | 0.86 |
| Middle adulthood | | | | | | |
| SOC | 0.94 | 0.95 | 0.97 | 0.93 | 0.92 | 0.95 |
| ES | 0.84 | 0.90 | 0.86 | 0.84 | 0.80 | 0.87 |
| LS | 0.84 | 0.91 | 0.86 | 0.84 | 0.80 | 0.87 |
| O | 0.86 | 0.93 | 0.87 | 0.85 | 0.81 | 0.88 |
| C | 0.84 | 0.93 | 0.87 | 0.84 | 0.80 | 0.87 |
| Late adulthood | | | | | | |
| SOC | 0.93 | 0.93 | 0.96 | 0.93 | 0.92 | 0.94 |
| ES | 0.82 | 0.90 | 0.84 | 0.82 | 0.78 | 0.85 |
| LS | 0.82 | 0.89 | 0.83 | 0.82 | 0.78 | 0.85 |
| O | 0.88 | 0.91 | 0.89 | 0.87 | 0.85 | 0.89 |
| C | 0.83 | 0.91 | 0.87 | 0.83 | 0.79 | 0.86 |

*Note:*

GLB – Greatest lower bound; SOC – the whole SOC48-PL Questionnaire; ES – Elective selection, LS – Loss-based selection; O – Optimization, C – Compensation.
