# Supplementary material for: Selection, optimization and compensation strategies and their relationship with well-being and impulsivity in early, middle and late adulthood in a Polish sample
Source: BMC Psychol. 2021 Sep 16;9:144. doi: 10.1186/s40359-021-00650-2 (PMC8447622; doi:10.1186/s40359-021-00650-2)
Supplement: Supplementary file 4 — Additional file 4. Table S2: Statistics for items of individual scales based on reliability analysis. Item-scale correlation coefficients and Cronbach's αwhen item is deleted for each item of particular SOC48-PL questionnaire scales [file 40359_2021_650_MOESM4_ESM.docx]

**Selection, optimization and compensation strategies and their relationship with well-being and impulsivity in early, middle and late adulthood in a Polish sample**

Ludmiła Zając-Lamparska^1^

^1^ Faculty of Psychology, Kazimierz Wielki University in Bydgoszcz, Poland

**Author Note**

Ludmiła Zając-Lamparska [
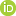
](https://orcid.org/0000-0003-4618-547X) https://orcid.org/0000-0003-4618-547X

Correspondence concerning this article should be addressed to Ludmiła Zając-Lamparska, Faculty of Psychology, Kazimierz Wielki University, ul. Staffa 1, 85-867 Bydgoszcz, Poland. Email: [lzajac@ukw.edu.pl](mailto:lzajac@ukw.edu.pl)

**Table 2**

*Statistics for items of individual scales based on reliability analysis*

| ES scale | | | LS scale | | | O scale | | | C scale | | |
| --- | --- | --- | --- | --- | --- | --- | --- | --- | --- | --- | --- |
| Item | CIS | AIE | Item | CIS | AIE | Item | CIS | AIE | Item | CIS | AIE |
| Whole sample | | | | | | | | | | | |
| ES1 | 0.35 | 0.80 | LS1 | 0.31 | 0.80 | O1 | 0.68 | 0.83 | C1 | 0.53 | 0.83 |
| ES2 | 0.45 | 0.79 | LS2 | 0.34 | 0.80 | O2 | 0.65 | 0.83 | C2 | 0.46 | 0.83 |
| ES3 | 0.54 | 0.78 | LS3 | 0.45 | 0.79 | O3 | 0.64 | 0.83 | C3 | 0.46 | 0.83 |
| ES4 | 0.33 | 0.80 | LS4 | 0.47 | 0.79 | O4 | 0.70 | 0.83 | C4 | 0.46 | 0.83 |
| ES5 | 0.55 | 0.78 | LS5 | 0.52 | 0.79 | O5 | 0.55 | 0.84 | C5 | 0.49 | 0.83 |
| ES6 | 0.51 | 0.78 | LS6 | 0.42 | 0.79 | O6 | 0.68 | 0.83 | C6 | 0.47 | 0.83 |
| ES7 | 0.54 | 0.78 | LS7 | 0.53 | 0.78 | O7 | 0.22 | 0.86 | C7 | 0.62 | 0.82 |
| ES8 | 0.59 | 0.78 | LS8 | 0.31 | 0.80 | O8 | 0.58 | 0.83 | C8 | 0.61 | 0.82 |
| ES9 | 0.44 | 0.79 | LS9 | 0.51 | 0.79 | O9 | 0.13 | 0.87 | C9 | 0.39 | 0.84 |
| ES10 | 0.44 | 0.79 | LS10 | 0.52 | 0.78 | O10 | 0.50 | 0.84 | C10 | 0.50 | 0.83 |
| ES11 | 0.17 | 0.81 | LS11 | 0.56 | 0.78 | O11 | 0.62 | 0.83 | C11 | 0.52 | 0.83 |
| ES12 | 0.51 | 0.78 | LS12 | 0.49 | 0.79 | O12 | 0.39 | 0.85 | C12 | 0.54 | 0.82 |
| Early adulthood | | | | | | | | | | | |
| ES1 | 0.29 | 0.78 | LS1 | 0.35 | 0.75 | O1 | 0.63 | 0.81 | C1 | 0.55 | 0.81 |
| ES2 | 0.41 | 0.77 | LS2 | 0.18 | 0.77 | O2 | 0.56 | 0.82 | C2 | 0.46 | 0.81 |
| ES3 | 0.49 | 0.76 | LS3 | 0.49 | 0.74 | O3 | 0.55 | 0.82 | C3 | 0.46 | 0.81 |
| ES4 | 0.25 | 0.78 | LS4 | 0.46 | 0.74 | O4 | 0.64 | 0.81 | C4 | 0.36 | 0.82 |
| ES5 | 0.53 | 0.76 | LS5 | 0.48 | 0.74 | O5 | 0.56 | 0.82 | C5 | 0.48 | 0.81 |
| ES6 | 0.48 | 0.76 | LS6 | 0.38 | 0.75 | O6 | 0.66 | 0.81 | C6 | 0.50 | 0.81 |
| ES7 | 0.47 | 0.76 | LS7 | 0.43 | 0.75 | O7 | 0.19 | 0.85 | C7 | 0.66 | 0.80 |
| ES8 | 0.61 | 0.75 | LS8 | 0.23 | 0.77 | O8 | 0.61 | 0.81 | C8 | 0.59 | 0.80 |
| ES9 | 0.37 | 0.77 | LS9 | 0.52 | 0.73 | O9 | 0.13 | 0.85 | C9 | 0.31 | 0.83 |
| ES10 | 0.44 | 0.77 | LS10 | 0.46 | 0.74 | O10 | 0.50 | 0.82 | C10 | 0.46 | 0.82 |
| ES11 | 0.24 | 0.79 | LS11 | 0.47 | 0.74 | O11 | 0.61 | 0.81 | C11 | 0.45 | 0.81 |
| ES12 | 0.48 | 0.76 | LS12 | 0.40 | 0.75 | O12 | 0.38 | 0.83 | C12 | 0.53 | 0.81 |
| Middle adulthood | | | | | | | | | | | |
| ES1 | 0.43 | 0.83 | LS1 | 0.42 | 0.83 | O1 | 0.66 | 0.82 | C1 | 0.57 | 0.82 |
| ES2 | 0.54 | 0.83 | LS2 | 0.50 | 0.82 | O2 | 0.68 | 0.82 | C2 | 0.41 | 8.83 |
| ES3 | 0.57 | 0.82 | LS3 | 0.48 | 0.83 | O3 | 0.65 | 0.82 | C3 | 0.52 | 0.83 |
| ES4 | 0.47 | 0.83 | LS4 | 0.52 | 0.82 | O4 | 0.68 | 0.82 | C4 | 0.49 | 0.83 |
| ES5 | 0.62 | 0.82 | LS5 | 0.63 | 0.81 | O5 | 0.43 | 0.84 | C5 | 0.52 | 0.83 |
| ES6 | 0.55 | 0.82 | LS6 | 0.53 | 0.82 | O6 | 0.70 | 0.82 | C6 | 0.41 | 0.83 |
| ES7 | 0.55 | 0.82 | LS7 | 0.53 | 0.82 | O7 | 0.20 | 0.86 | C7 | 0.60 | 0.82 |
| ES8 | 0.61 | 0.82 | LS8 | 0.39 | 0.83 | O8 | 0.57 | 0.83 | C8 | 0.55 | 0.82 |
| ES9 | 0.42 | 0.83 | LS9 | 0.44 | 0.83 | O9 | 0.14 | 0.86 | C9 | 0.38 | 0.84 |
| ES10 | 0.45 | 0.83 | LS10 | 0.48 | 0.83 | O10 | 0.45 | 0.84 | C10 | 0.48 | 0.83 |
| ES11 | 0.21 | 0.85 | LS11 | 0.62 | 0.82 | O11 | 0.61 | 0.83 | C11 | 0.57 | 0.82 |
| ES12 | 0.60 | 0.82 | LS12 | 0.45 | 0.83 | O12 | 0.43 | 0.84 | C12 | 0.54 | 0.82 |
| Late adulthood | | | | | | | | | | | |
| ES1 | 0.40 | 0.81 | LS1 | 0.32 | 0.81 | O1 | 0.73 | 0.85 | C1 | 0.49 | 0.82 |
| ES2 | 0.52 | 0.80 | LS2 | 0.31 | 0.82 | O2 | 0.71 | 0.85 | C2 | 0.47 | 0.82 |
| ES3 | 0.55 | 0.80 | LS3 | 0.44 | 0.80 | O3 | 0.71 | 0.85 | C3 | 0.41 | 0.82 |
| ES4 | 0.30 | 0.82 | LS4 | 0.48 | 0.80 | O4 | 0.75 | 0.85 | C4 | 0.45 | 0.82 |
| ES5 | 0.52 | 0.80 | LS5 | 0.55 | 0.79 | O5 | 0.61 | 0.86 | C5 | 0.46 | 0.82 |
| ES6 | 0.53 | 0.80 | LS6 | 0.44 | 0.80 | O6 | 0.69 | 0.85 | C6 | 0.47 | 0.82 |
| ES7 | 0.56 | 0.80 | LS7 | 0.60 | 0.79 | O7 | 0.27 | 0.88 | C7 | 0.61 | 0.81 |
| ES8 | 0.58 | 0.80 | LS8 | 0.32 | 0.81 | O8 | 0.61 | 0.86 | C8 | 0.61 | 0.80 |
| ES9 | 0.50 | 0.81 | LS9 | 0.53 | 0.80 | O9 | 0.16 | 0.88 | C9 | 0.33 | 0.83 |
| ES10 | 0.46 | 0.81 | LS10 | 0.56 | 0.79 | O10 | 0.54 | 0.86 | C10 | 0.50 | 0.81 |
| ES11 | 0.24 | 0.83 | LS11 | 0.54 | 0.80 | O11 | 0.60 | 0.86 | C11 | 0.53 | 0.81 |
| ES12 | 0.52 | 0.80 | LS12 | 0.51 | 0.80 | O12 | 0.38 | 0.87 | C12 | 0.50 | 0.81 |

*Note:*

CIS – correlation item-scale ; AIE – alpha when item is deleted; ES – Elective selection, LS – Loss-based selection; O – Optimization, C – Compensation
