# Supplementary material for: Selection, optimization and compensation strategies and their relationship with well-being and impulsivity in early, middle and late adulthood in a Polish sample
Source: BMC Psychol. 2021 Sep 16;9:144. doi: 10.1186/s40359-021-00650-2 (PMC8447622; doi:10.1186/s40359-021-00650-2)
Supplement: Supplementary file 5 — Additional file 5. Table S3: Pearson's correlations coefficients between the results obtained during the first test and retest in particular items of the SOC48-PL questionnaire. Correlation coefficients (Pearson's r) for individual SOC48-PL questionnaire items obtained in the test-retest reliability evaluation procedure [file 40359_2021_650_MOESM5_ESM.docx]

**Selection, optimization and compensation strategies and their relationship with well-being and impulsivity in early, middle and late adulthood in a Polish sample**

Ludmiła Zając-Lamparska^1^

^1^ Faculty of Psychology, Kazimierz Wielki University in Bydgoszcz, Poland

**Author Note**

Ludmiła Zając-Lamparska [
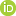
](https://orcid.org/0000-0003-4618-547X) https://orcid.org/0000-0003-4618-547X

Correspondence concerning this article should be addressed to Ludmiła Zając-Lamparska, Faculty of Psychology, Kazimierz Wielki University, ul. Staffa 1, 85-867 Bydgoszcz, Poland. Email: [lzajac@ukw.edu.pl](mailto:lzajac@ukw.edu.pl)

**Table 3**

7

*Pearson’s correlations coefficients between the results obtained during the first test and retest in particular items of the SOC48-PL questionnaire*

| ES scale | | LS scale | | O scale | | C scale | |
| --- | --- | --- | --- | --- | --- | --- | --- |
| ES1 | 0.63 | LS1 | 0.55 | O1 | 0.69 | C1 | 0.58 |
| ES2 | 0.70 | LS2 | 0.56 | O2 | 0.71 | C2 | 0.72 |
| ES3 | 0.63 | LS3 | 0.55 | O3 | 0.69 | C3 | 0.74 |
| ES4 | 0.58 | LS4 | 0.62 | O4 | 0.69 | C4 | 0.63 |
| ES5 | 0.75 | LS5 | 0.62 | O5 | 0.63 | C5 | 0.68 |
| Es6 | 0.68 | LS6 | 0.51 | O6 | 0.68 | C6 | 0.60 |
| ES7 | 0.60 | LS7 | 0.70 | O7 | 0.70 | C7 | 0.66 |
| ES8 | 0.70 | LS8 | 0.60 | O8 | 0.64 | C8 | 0.73 |
| ES9 | 0.77 | LS9 | 0.68 | O9 | 0.65 | C9 | 0.57 |
| ES10 | 0.59 | LS10 | 0.64 | O10 | 0.72 | C10 | 0.57 |
| ES11 | 0.66 | LS11 | 0.53 | O11 | 0.69 | C11 | 0.68 |
| ES12 | 0.62 | LS12 | 0.65 | O12 | 0.64 | C12 | 0.59 |

*Note:*

ES – Elective selection, LS – Loss-based selection; O – Optimization, C – Compensation

All correlations coefficients are statistically significant at *p* < .001, and at *q* < .001 after application of FDR correction for multiple comparisons
